# Determination of Interface Fracture Parameters in Thermoplastic Fiber Metal Laminates Under Mixed-Mode I+II

**DOI:** 10.3390/polym17111462

**Published:** 2025-05-24

**Authors:** Michał Smolnicki, Szymon Duda

**Affiliations:** Faculty of Mechanical Engineering, Wroclaw University of Science and Technology, 50-370 Wroclaw, Poland; szymon.duda@pwr.edu.pl

**Keywords:** FML, fracture energy, mixed-mode, FEA

## Abstract

Thermoplastic fiber metal laminates (FMLs) are hybrid material systems that consist of a thin aluminum alloy sheet bonded to plies of fiber-reinforced adhesive. They provide excellent properties like fatigue strength, damage-tolerant properties, and inherent resistance to corrosion. However, they are still challenging materials in terms of the metal–composite interface, which is the weakest link in this material system. In this paper, an experimental–numerical method for the determination of the fracture stress and energy for metal–composite interlayer is presented and verified. The proposed method utilizes four different experimental tests: DCB test (interface opening—mode I), ENF test (interface shearing—mode II), MMB test (mixed-mode I+II—opening with the shearing of the interface) and three-point bending test (3PB). For each test, digital twin in the form of a numerical model is prepared. The established numerical models for DCB and ENF allowed us to determine fracture stress and energy for mode I and mode II, respectively. On the basis of the numerical and experimental (from the MMB test) data, the B-K exponent is determined. Finally, the developed material model is verified in a three-point bending test, which results in mixed-mode conditions. The research is conducted on the thermoplastic FML made of aluminum alloy sheet and glass fiber reinforced polyamide 6. The research presented is complemented by fundamental mechanical tests, image processing and Scanning Electron Microscopy (SEM) analysis. As an effect, for the tested material, fracture parameters are determined using the described method.

## 1. Introduction

Layered material systems are widely used in various industries, providing superior properties, i.e., high relative strength, excellent fatigue and fracture characteristics, and high stiffness. An example of such material systems is fiber–metal laminates (FMLs), developed at Delf University, Netherlands, in the early 1980s [1]. Combining various materials, i.e., fiber-reinforced and metallic layers, can reveal many benefits, including greater resistance against rapid crack growth propagation compared to monolithic materials. The fracture properties of these materials are crucial to determine, especially since the bonding connection of various materials is the weakest link in the material system. Due to the possibility of recycling, thermoplastic materials have recently replaced the conventional thermoset materials used as the matrix in the mentioned hybrid layered systems. However, considering the structural application, thermoplastic materials still require a comprehensive investigation of the fracture properties, especially if the aerospace industry is concerned.

Several standardized mechanical techniques are devoted to determining the interface’s fracture properties using a scalar parameter, the strain energy release rate. Considering the fracture mechanics, three fracture modes are defined concerning the load direction acting on the crack, i.e., mode I—opening, mode II—in-plane shearing, and mode III—out-of-plane shearing. Mechanical tests for investigating the particular modes and their combination are listed in Table 1. Still, there is no standard for investigating mode III due to the challenges in providing the proper load conditions.

**Figure 1:**
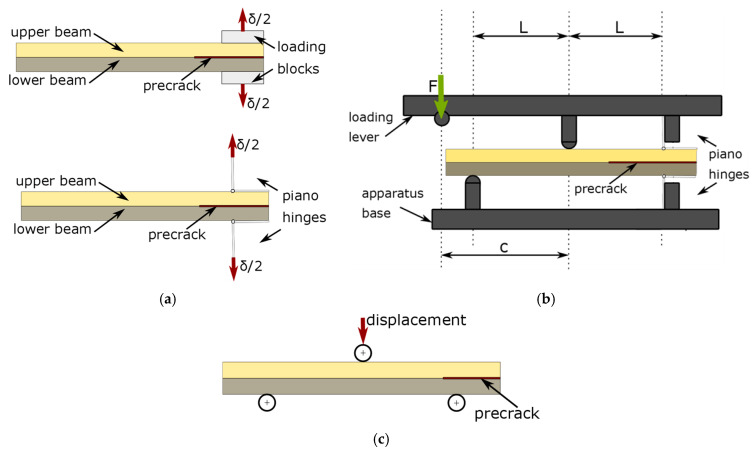
The schematic idea of loading conditions applied to investigate the interface fracture energy for (**a**) mode I-DCB, (**b**) mixed-mode I+II-MMB, and (**c**) mode II-ENF.

The schematic idea of listed mechanical tests is shown in Figure 1. The double cantilever beam (DCB) test shown in Figure 1a provides the loading conditions in mode I for flat specimens with precrack, whereby mode II is investigated by the setup shown in Figure 1b for similar sample geometry, i.e., flat laminate with precrack. The fracture examples of the mechanical objects show that the cracks are subjected to mixed-mode loading conditions, which can be mechanically investigated by the setup given in Figure 1c. Based on highlighted mechanical tests, the delamination can be described by strain energy release rates and applied along with the numerical methods for predicting the mechanical performance of laminated material systems under various load conditions.

The presented ASTM standards are valid only for unidirectional fiber-reinforced polymer matrix composite laminates, excluding the other configurations of laminates, including FMLs. The available research still includes the uncertainty in defining the energy release rate of newly hybrid materials. The authors already investigated thermoplastic materials developed at TU Chemnitz [6,7], providing a numerical–mechanical approach for some material configurations in [8,9,10,11]; however, the generalization of the developed method still requires a comprehensive study on various material sets and loading conditions. Also, in the aforementioned research papers, the authors were focused on separate modes. In the presented paper, a comprehensive approach is proposed and analyzed on the exemplary thermoplastic FML configuration.

The literature survey provided several scientific manuscripts dealing with the fracture properties of the interface layer in laminated materials. In Ref. [12], the mechanical characteristics of novel thermoplastic FMLs were conducted, showing outstanding in-plane mechanical characteristics compared to thermoset laminates. The applied manufacturing technology was conducted at room temperature, which reduces the residual thermal stresses. This research shows the potential of thermoplastic-based laminates, especially FMLs, in modern applications, providing comparable mechanical properties to the thermoset-based materials; however, this increases the recycling possibility of those materials.

Interlaminar fracture toughness was investigated in Ref. [13], and the strain energy density in fracture mode II for carbon-reinforced aluminum (CARALL) and glass-reinforced aluminum (GLARE) laminates was calculated using the developed analytical approach based on enhanced beam theory, and verified by the standardized compliance calibration method. The finite element method was applied to support performed experimental tests. The results show good agreement in calculating the energy release rate in mode II and might be important in order to increase accuracy in delivering this energy parameter; however, the research is limited to fracture mode II only. A comprehensive approach to investigating the FML joints was given in [14], where the digital image correlation (DIC), acoustic emission (AE), and numerical analysis (FEA) were applied to investigate the fracture behavior of the hybrid laminate. The mechanical performance under uniaxial tension was investigated; however, the research involves a complex numerical simulation incorporating the user subroutine including the 3D Hashin criterion, traction–separation characteristics, and ductile damage criterion; this allows for predicting the progressive damage of GFRP layers, bonding layers, and metal layers. The research shows the impact of geometrical parameters on the damaged nature of the joints and the effectiveness of numerical analysis incorporating advanced user subroutines in assessing the damage of laminated materials. Another example of incorporating numerical analysis for investigating adhesive bonded joints under mode I is presented in Ref. [15]. It shows the capability of the cohesive elements to assess the fracture properties of the interface in laminates. Nonetheless, the investigation of the fracture behavior should be complemented by the progressive damage failure to reflect the mesoscale failure nature of the laminate. The progressive model is an objective of Ref. [16]; it allows for assessing damage in laminates with arbitrary ply orientations and predicting the ultimate tensile strength of the notched laminates, showing a very good agreement with the experimental results. The complex application of the numerical model with cohesive zones and progressive damage is given in Ref. [17]. The prediction of the flexural behavior is investigated within Ref. [18]; however, the adhesive layer in this case was ignored due to the lack of slip between layers. Although the presented results show reasonable agreement, the post-failure form indicates that including the cohesive behavior might positively impact the obtained results. Besides the prediction of fracture behavior of the adhesive interface, the improvement in the bonding connection is an important aspect investigated in Ref. [19], where several toughening methods were applied including acid etching, surface treatment, and nanofiller addition. The DCB test along with the numerical model was applied to assess the impact of the methods, and only filler additions decreased the interlaminar strength due to difficult dispersion in the matrix material. Other methods and their combinations positively impacted the interface’s mechanical performance. In their work, (Ref. [20]) Zhou and Huang proposed an alternative approach to creating a numerical model of delamination in hybrid laminate by introducing stress modification based on DCB and ENF tests and utilizing the UMAT subroutine. They achieve a good correlation of the numerical model with an experimental test of U-shape drawing.

**Figure 2:**
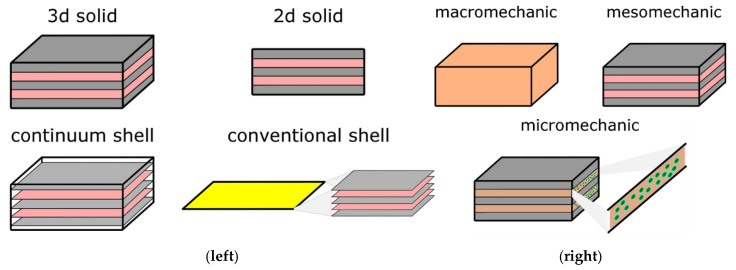
Types of numerical models based on the dimensionality (**left**) and material homogenization level (**right**). Graphics made by the author for the purpose of [21].

An important aspect of the presented research is utilizing numerical simulations realized with finite element method. The authors devoted to this issue a comprehensive review titled “A Review on Finite-Element Simulation of Fibre Metal Laminates” [21] which in detail explains important factors about it. Because of that, in this introduction, we will mention only a few important points, and other aspects can be found in the aforementioned review paper. Modeling failure in FMLs using FEA requires building a proper numerical model. To achieve this, one has to address crucial issues like the dimensionality of the model, which can be 3D solid (the most popular choice, e.g., [22,23]), or sometimes other options like 2D solid [24], 2D shell [17] or even 2D continuum shell [25]. Another important issue is the definition of the material model, or more precisely, the level of its homogenization. The material can be defined for the whole material at once (macromechanics approach), for each of the layers separately (mesomechanic) and finally with distinguishing every single fiber and matrix (micromechanic). While the macromechanics approach can be good enough to capture the elastic behavior of the material [8,26], it is not sufficient for the failure prediction stage and also is not able to capture interface behavior. A micromechanical approach can be used for obtaining data for other approaches [12], but it is too complex for large-scale analysis. Therefore, for the numerical simulations in the scope of this paper, a mesomechanical approach is used. Different approaches to the dimensionality and the material homogenization are presented in Figure 2.

The failure in the FML can be split into two aspects—the behavior of layers and the behavior of interfaces. For the layers, classic criteria for respective materials are used which can include, e.g., maximum principle stress criterion for metals and matrices and classic composite criteria like interactive ones like Puck [27] or Tsai-Wu [28], or separate mode criteria like Hashin or Hashin-Rotem [29,30]. However, in this paper, the main focus is on the interface between metal and composite. The most commonly [23,31,32] utilized approach here is to use the Cohesive Zone Model (CZM) first proposed by Dugdale [33] and Barenblatt [34]. The numerical software usage of this approach requires a definition of the so-called traction–separation law, which can have various forms like triangular or trapezoid. In this paper, we show the method of determining parameters for such laws using an experimental–numerical approach. For the singular mode of failure, the law has a shape of function:

$$\sigma =f\left(\delta \right)$$

In the most common triangular form, it requires the user to define two parameters of maximum stress, i.e., $\sigma_{IC}$ and either fracture energy $G_{IC}$ or critical displacements. However, if we consider mixed-mode loading conditions, defining such laws for each separate mode is not sufficient. We need to formulate another formula that will be able to predict TSL for any mixicity mode. Widely used in this regard is the Benzeggah and Kenane approach (B-K) [35], which is given for mixed-mode I+II by the following formula:

$$G_{TC}=G_{IC}+\left(G_{IIC}-G_{IC}\right)\cdot {\left(\frac{G_{II}}{G_{I}+G_{II}}\right)}^{m}$$

Another option for this issue is the so-called power-law, which also is sometimes utilized in research [23]. Both can be used in the proposed method, but the exemplary configuration will be analyzed using the B-K approach.

Highlighted manuscripts show the progress in investigating the layered hybrid materials using numerical methods; however, supplementation in this field is required in terms of the mechanical performance of the adhesive interface itself if it is the weakest link of a material system. There is significant research performed in the field; nonetheless, the addition of the mixed mode will impact our understanding of the complex behavior of the interface.

The presented paper proposes a method for delivering the metal–composite interface fracture properties by utilizing both experimental and numerical approaches with a specific set of tests. In numerical modeling, it applies the cohesive zone model for assessing the fracture properties of newly developed FML with a thermoplastic matrix, considering in particular the metal–composite bonding interface. Due to the material architecture, the available standards can only be followed in a limited way; however, they provide fundamentals for investigating the fracture properties under modes I, and II and their combination. The traction–separation characteristics and strain energy release rate are applied to reflect the behavior of the interface. The created discrete model is assessed by a 3-point bending test. Effect parameters for traction–separation law with B-K coefficient are obtained for tested material and verified with separate tests. The method proposed in this paper can be generalized to other similar materials where the interface between the metal and composite layer has to be assessed.

## 2. Experimental Program

This section focuses on the mechanical testing of material in order to obtain the data for building numerical models and validating them. In an experimental campaign, three stages can be distinguished:

- Assessment of constituents (mechanical properties of metal and composite components)
- Assessment of fracture behavior of metal–composite interface in manufactured fiber metal laminate (DCB, ENF, MMB)
- Assessment of fracture behavior in test with variable mixicity-mode (3PB) for validation of the obtained material model

### 2.1. Material and Manufacturing

**Figure 3:**
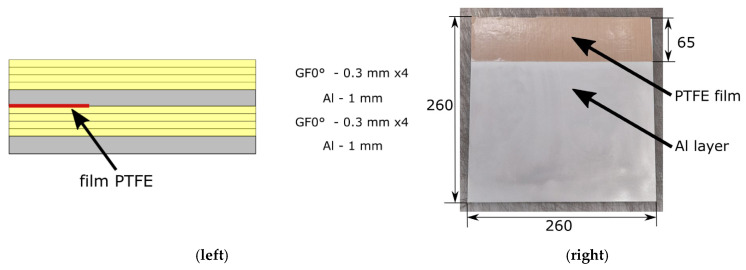
Material configuration used in the research (**left**) stacking sequence is shown (chosen to ensure equal stiffness in upper and lower beams. In the case of the 3PB specimens without PTFE were used; (**right**) main dimension of the manufactured plate is shown with the positioning of the PTFE layer.

The proposed method was tested on FML characterized using aluminum alloy AW-6061 T6 as a metal layer and PA6 reinforced by glass fibers (by utilizing prepreg Celstran PA6-GF60-01—produced by Celanese, Irving, TX, USA). The designed material is characterized by an Al—GF 0° interface, which will be the main subject of the research. The number of layers was chosen based on the desired total thickness for mechanical tests which is between 3 and 5 mm (here the total thickness was planned to be 4.4 mm). For all the tests that require initial delamination, PTFE film was planned to be introduced into the material. Specific asymmetrical lay-up configuration $\left[0{{}^{\circ}}_{4}/Al/0{{}^{\circ}}_{4}/Al\right]$ was chosen because of the similar stiffness in both parts separated by PTFE film—which is important for DCB and MMB tests. Figure 3 demonstrates this sequence in graphical form.

**Figure 4:**
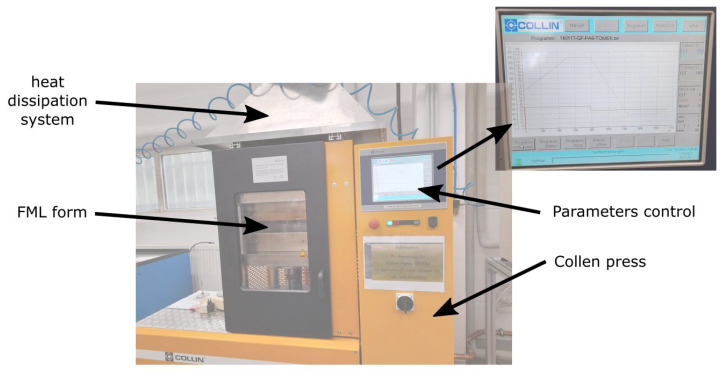
Collen plate press with temperature control. FMLs were bonded in a 20-min long process that includes temperature (up to 280 °C) and pressure (35 bar).

The material was manufactured in the form of plates with dimensions 260 mm × 260 mm using the hot-press technology with a temperature of 280 °C under 17.5 bar of pressure. After cooling, the curing process was continued at room temperature—this process was optimized for GF and PA6 during previous stages of research. Aluminum sheets and composite prepreg were cut to the desired dimensions and then stacked according to the lay-up configuration presented in Figure 3 (left). The principal dimensions and placement of PTFE film are presented in Figure 3 (right). The bonding process was realized using the hot-press technique. The pressure and the temperature were provided using plate press (Labor Plattenpresse P 300 PM, power 29 kW, produced by Collin, Maitenbeth, Germany). The plates were pressed for a total time of 20 min, which included 5 min at a maximum temperature of 250 °C and 35 bar of pressure. The manufacturing setup is shown below in Figure 4.

A total of four plates were manufactured for this research, including two plates with PTFE film and two without it. Plates were then cut into specimens for particular tests. All specimens were designed to have the same width of 20 mm. The length varied between 160 and 200 mm and was chosen based on standards for tests that we were using [4,5,36]. Although they cannot be followed literally since they are designed for unidirectional composites and not for FMLs, we decided to follow their general recommendation in the area of the specimen size as well as experiment conditions wherever it was possible (limitations are caused by different materials in the research and in the aforementioned standards). The summary of specimen dimensions used in the research is presented in Table 2.

**Figure 5:**
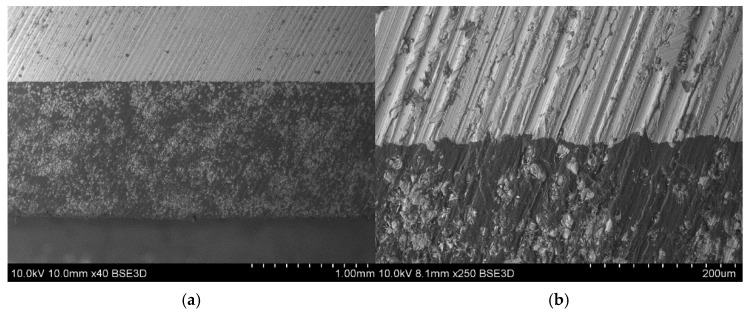
SEM images of manufactured laminates: (**a**) 40× zoom—visible uneven distribution of the fibers in composite layer (**b**) 250× zoom—visible good bonding between composite and metal layer.

The quality of manufacturing was assessed by fractography investigation realized with scanning electron microscopy (SEM). SEM images (examples shown in Figure 5) show good bonding between metal and composite later achieved in the manufacturing process. Based on the SEM images and their histograms, the fiber content was determined using Python 3.10 script as ~60%. The method utilizes the fact that, in the SEM images, fibers and matrices have distinct colors. Images are processed using filters like Gaussian blurring and then recalculated from grayscale to black and white picture. The picture is further processed to remove imperfections. Fiber content is then calculated as the ratio of white and black pixels. The method applied was described by the authors more widely in [37]. The value obtained was along with the producer of the prepreg declaration.

### 2.2. Tensile Test—Material and Constituent Assessment

**Figure 6:**
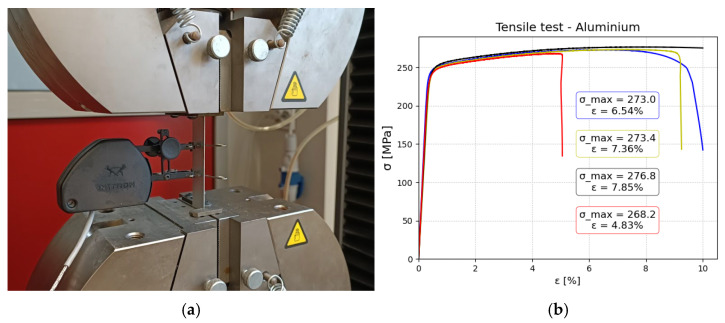
Aluminum tensile tests: (**a**) experimental setup with extensometer for measuring strains (**b**) stress–strain relationship obtained for four specimens.

**Figure 7:**
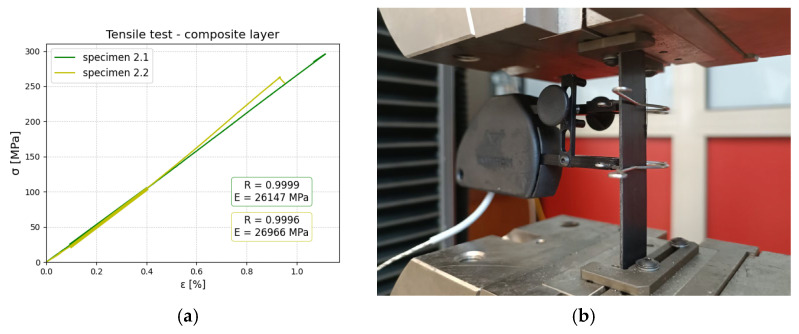
Composite (PA6 reinforced with glass fibers) tensile tests: (**a**) stress–strain relationship obtained for composite 0° layers (**b**) experimental setup with extensometer for measuring strains.

**Figure 8:**
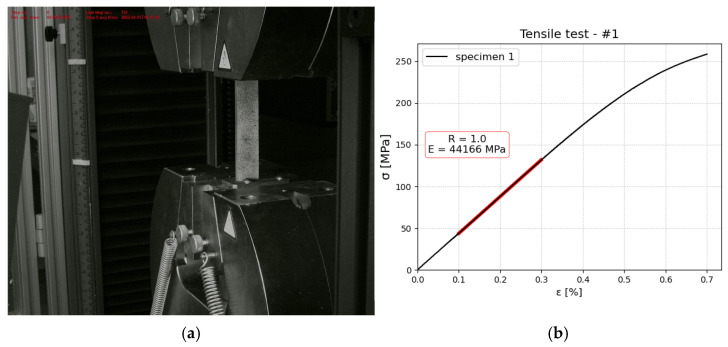
FML exemplary tensile tests: (**a**) experimental setup with DIC system for measuring strains—the specimen is coated with black and white pattern (**b**) stress–strain relationship obtained for specimen 06D.

Tensile tests were conducted for constituents of aluminum and composite (in two directions), as well as for the manufactured FML. The main reason was to obtain reliable elastic material data for numerical modeling. Especially in the case of composite layers during the manufacturing process, we observed a flash of the matrix material, which implies that the real properties of composites will be different from the theoretical one, as the fiber content will be higher than 60%. All tests in this section were conducted using an INSTRON 8592 machine (produced by Instron, Norwood, MA, USA). Tests were realized with a constant speed of v=0.5 mm/min. From the tensile test, Young’s modulus was determined utilizing linear regression using Python script. Below, the results and experimental setup are shown for aluminum (Figure 6), polyamide 6 reinforced with glass fibers (Figure 7), and FML (Figure 8). In the first two cases, strains were measured with an extensometer with a measurement base of 25 mm. Stress was calculated as a ratio of tensile force and cross-section area. The thickness of aluminum and composite was measured as 1 and 1.1 mm, respectively. In both cases, the width was measured as 20.3 mm.

Based on the investigation, the following material data were obtained:

- Aluminium: E = 68,713.4 ± 4311 MPa
- Composite layer-only fibers parallel to the length of the specimen E1 = 26,556 ± 579 MPa, E2 = 4846.5 ± 12 MPa

**Figure 9:**
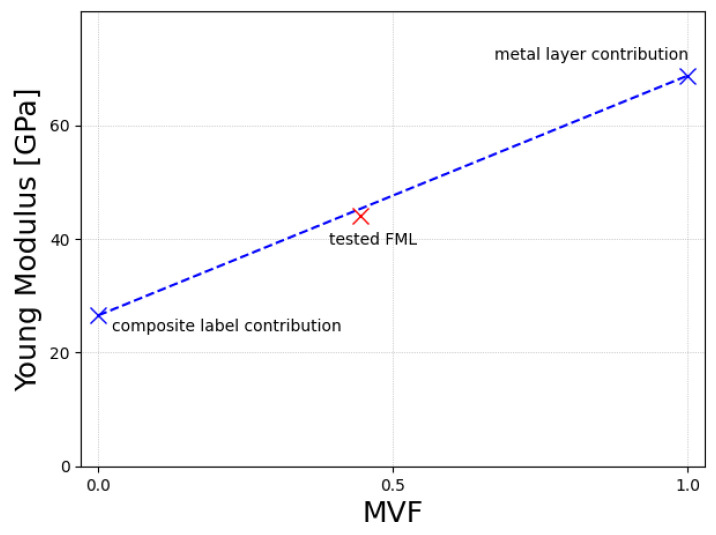
Metal volume fraction analysis for the researched material. Blue dashed line connects the Young modulus obtained for the metal and composite constituent alone. With the red marker, we denoted the tested value for the FML with MVF = 44.(4).

Analysis of the constituent data and FML tested under tensile load using the Metal Volume Fraction (MVF) approach shows a good correlation between all experimental tests. A small overestimation of the Young modulus using the MVF method can be explained by minor slippages that were observed during the FML test. The analysis is presented in graphical form in Figure 9.

### 2.3. Experimental Campaign—DCB, ENF, MMB and 3PB Tests

The main experimental campaign was conducted on manufactured FML material using specimens as described in Table 2. The goal of it is to determine the fracture properties of composite–metal interfaces with the support of numerical simulations. This part of the research consisted of four different types of tests:

- DCB test (double cantilever beam test) realized on specimens with initial delamination for testing interface strength in mode I (opening of the interface)
- ENF test (end-notch flexural test) realized on specimens with initial delamination for testing interface strength in mode II (shearing of the interface)
- MMB test (end-notch flexural test) realized on specimen with initial delamination for testing interface strength in mixed-mode I+II (opening and shearing of the interface)
- 3PB test (three-point bending test) realized on specimen without initial delamination for testing the overall behavior of the material including interface strength

The idea behind the choice of such tests is that the DCB test can provide data about the mode I traction–separation relationship and the ENF test for mode II. The MMB test is used to determine parameters necessary for models, determining parameters in mixed-mode I+II based on characteristics for singular mode I and mode II. Finally, because values will be determined with the numerical models, the 3PB test is planned as validation data, which will enable checking if the model determined from DCB, ENF, and MMB is correct and can be used in the numerical analysis of cases with complex geometry.

**Figure 10:**
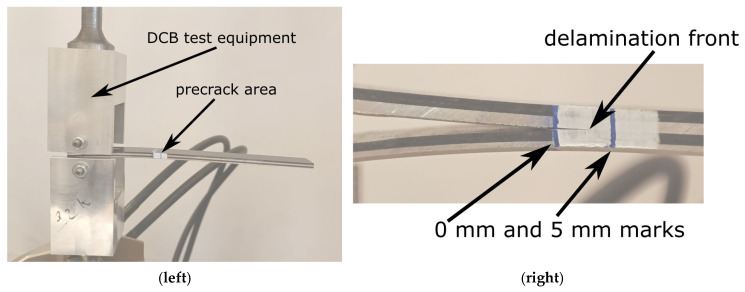
DCB test setup before the precrack stage (on the **left**) and a close-up of the precrack area with 0 and 5 mm marks on the white layer during the precrack stage (on the **right**).

Standards for DCB and ENF tests are designed for materials like CFRP and GFRP and cannot be used for FMLs directly. However, in areas where it was possible (mostly equipment, and sizes), we followed standard [2]. Precracking of DCB and ENF specimens was realized using the Bionix universal testing machine (produced by MTS, Eden Prairie, MN, USA). Each specimen was loaded until a crack with a length between 3 mm and 5 mm occurred. By inducing precrack, it is assured that the radius of the crack front coincides with 0. To enhance visual examination of the crack length, white paint layers were made on the rear face of the specimens, with the blue marking indicating 0 and 5 mm. The experimental setup and close-up of the precrack are presented in Figure 10. An analogical setup was used in the proper test with the exception of the universal testing machine which was changed to Instron 5944 (produced by Instron, Norwood, MA, USA). The change was made as the latter is an electric machine with a much lower background noise level during the test and also was equipped with ±2 kN force gauge, providing more accuracy for the investigated force range. Thanks to the design of a composite lay-up in the way that both beams (lower and upper) had similar stiffness, the experiment was conducted without problems.

The ENF test was realized also on Instron 5944 similar to the previously described procedure of preparing precrack on the specimen for the DCB test. Similarly to the DCB test, there is ASTM standard D7905 defined for unidirectional composites describing the determination of the mode II interlaminar fracture toughness of unidirectional fiber-reinforced polymer matrix composites [4]. For the same reasons, it cannot be followed entirely, especially in the determination of the fracture energy part, but we utilize the proposition of the specimen and test setup. Specimen dimensions used in this part of the research are described above in Table 2. The experimental setup consisted of two supports and one indenter. A summary of the most important parameters of the test is presented in Table 3.

**Figure 11:**
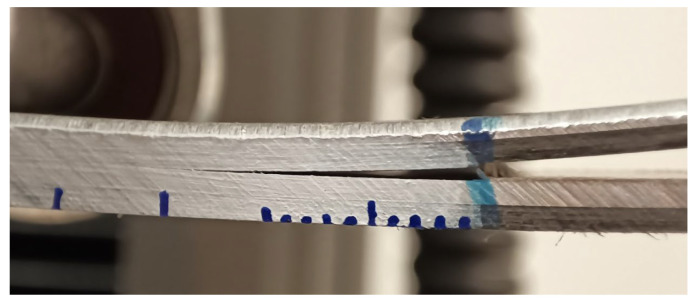
Close-up of specimen—rear face is covered with white paint and marked with blue dashed for easier recognition of the current crack length.

The mixed-mode I+II crack loading test was realized using the setup and equipment proposed for unidirectional fiber-reinforced polymer matrix composites in ASTM standard D6671M (MMB test) [5]. Similarly, to the DCB and ENF tests, it cannot be used for the calculation of fracture energy directly, but we utilize equipment, experimental setup, and specimen suggestions. Tests were realized on Instron 5944 due to smaller noise in comparison with hydraulic machines and adjusted range of force (up to 2kN). Specimens were coated with white paint and marked with blue dashes for easier recognition of progressing delamination. A general view of the setup and close-up of a specimen with delamination is presented in Figure 11. In the figure plastic deformation of aluminum is visible, which indicates that any approach that calculates energies based on the force–displacement dependency cannot be used. That is the reason why we propose another approach in place of the one proposed for the unidirectional composites in the ASTM standards.

The MMB test was chosen as it ensures a constant mixicity ratio ϕ during the whole test. Mixicity ratio was regulated by setting lever distance c. The main goal of these tests was to find the material coefficient m required for the Benzeggah–Kenane equation describing mixed-mode behavior. During interface fracture, two different lever lengths were utilized:

- c = 69.5 mm, which reflects mixicity ratio ϕ=33%
- c = 52 mm, which reflects mixicity ratio ϕ=45.5%

Values shown above for c were calculated using the formula written below, where L is the length of the MMB specimen between supports:

$$c=\frac{L\left(0.5\sqrt{3\cdot \frac{1-\phi }{\phi }}+1\right)}{3-0.5\sqrt{\frac{3\left(1-\phi \right)}{\phi }}}$$

For each case, a total of six specimens were tested. These specimens had problems with adhesion between piano hinges and specimens were excluded from further examination as not valid.

**Figure 12:**
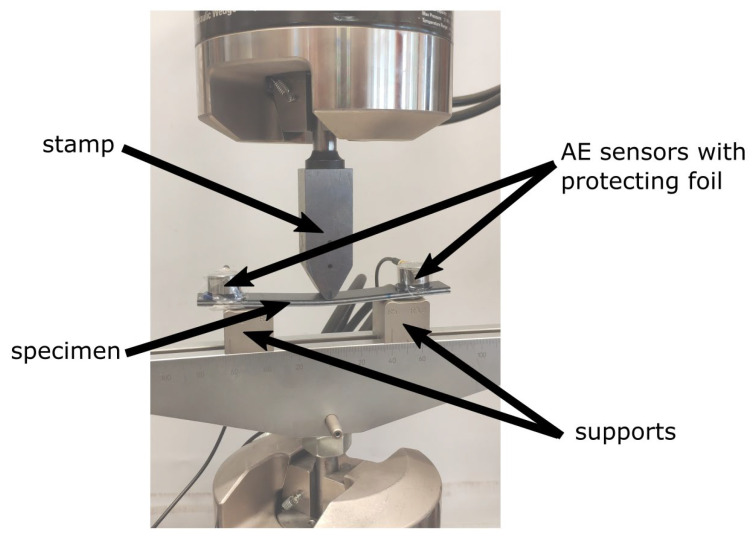
Experimental setup for the three-point bending test. On the specimen, two AE sensors are placed and protected by using foil for the possibility of rupture fracture of the specimen.

Finally, a three-point bending test was realized to validate the material model built based on previous experimental tests and numerical models. For the three-point bending test, specimens without initial delamination were used. Specimen dimensions used in this part of the research are described above in Table 2. Important parameters of this test are presented in Table 4. Tests were realized on the MTS Bionix universal testing machine, as predicted forces were over 2 kN. An overview of the experimental stand is presented in Figure 12.

### 2.4. Numerical Modelling

In the presented research, digital twins for each conducted experimental test were created using the Simulia Abaqus 6.14 environment. In the case of tensile, DCB, ENF, and MMB the goal was to find values of parameters that reflect these measured during experiments. The determined material model was then used on the 3PB model and validated with the experiment to confirm that the obtained model was correct. All numerical models were created in three-dimensional space using solid elements. The main reason behind this was that it enables the inclusion of effects that are not uniform along the thickness as delamination. Geometry was modeled using dimensions measured from specimens that were used in the experimental campaign. Material properties were designated to each layer separately (mesoscopic) approach. The data were based on experimental testing as described in the previous section and the missing data like Poisson coefficients for the composite layer were calculated using a micromechanics approach with eLamX^2^ software version 2 provided by TU Dresden [38] Because prepreg was used in the manufacturing process, we did not have exact information about the type of the matrix or fibers, so we assumed typical values for the glass fibers ($E_{II}$ = 75,000 MPa, ν=0.23, G=30,500 MPa) and PA6 (E = 2700 MPa, ν=0.391, G=970 MPa). Using the mentioned software for micromechanics, we determined values of $E_{\left|\right|}=46\;\mathrm{GPa},\;\nu =0.3$ and G=2315  MPa. However, the material was not ideal due to the method of manufacturing which leads to lower values than those calculated using micromechanical models. For example, this calculated value of Young’s modulus was much higher than one extracted by us from the tests ($E_{micro}=46\;\mathrm{GPa}$ versus $E_{test}=26.5\;\;\mathrm{GPa})$. Analysis of similar material data available shows that test-based value is more realistic (see [39]), so in the numerical models, we also used a lower value for the two Shear moduli than initially calculated (1800 GPa). In the case of the interface, it was assumed that it had elastic properties of PA6 and is modeled using elastic traction in Abaqus.

For models reflecting DCB, ENF, and MMB tests, the metal–composite interface was modeled using cohesive elements with non-zero thickness. Thickness was assumed to be of a PTFE film, which was confirmed by optical analysis. Cohesive elements had PA6 elastic properties (assumed based on material card E = 2700 MPa, G = 970 MPa) assigned to them, and their degradation was realized through the cohesive zone model (CZM). In the research, a bilinear version of the traction–separation law describing the cohesive zone was used. In the case of the DCB and ENF tests, values of initiation and fracture energy were chosen in a way that fits the experimental results. In the case of the MMB test, values from DCB and ENF models were used for mode I and mode II properties, respectively, and the Benzeggah–Kenane (B-K) exponent parameter was chosen in a way that satisfied experimental results. In the case of three-point bending, previously determined material parameters were used, and the results were compared with experimental results to validate the material model. The interface between composite layers was assumed to be perfect (as the strength of the metal–composite interface is much lower). The numerical model developed for the DCB test digital twin consists of linear hexahedral elements of type C3D8R, with cohesive element size set to 0.15 mm. The size of cohesive elements was determined by analyzing different values, and is a compromise between the computational time and accuracy. The simulation was displacement controlled through the reference points (connected to the model by kinematic coupling) and the forces were obtained from the results as reaction forces, as this approach enables registering force loss during the test, which is necessary for the analysis of composites (as the force can drop and then rise again due to structure, e.g., lever length increase and additional effects like fiber-bridging). The metal layers were modeled as isotropic materials, while composite layers were defined as orthotropic materials. For these materials, values of engineering constants were obtained from tensile testing as shown in the subchapter titled Tensile test—material and constituent assessment.

**Figure 13:**
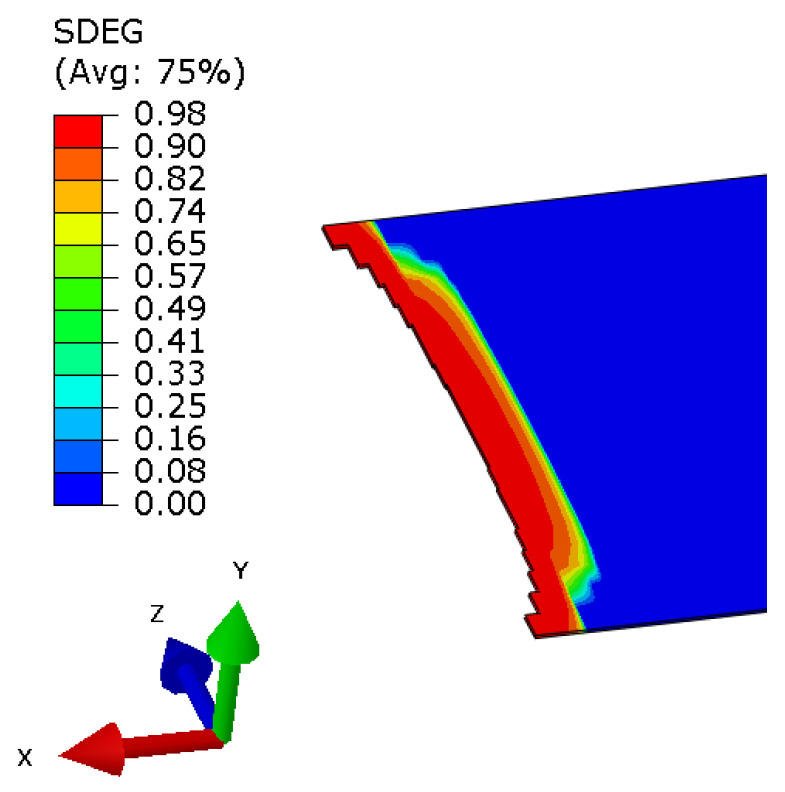
Delamination in DCB is shown as a cohesive elements layer with removed elements. The front crack is not linear.

Utilization of a three-dimensional solid model and cohesive elements enables analysis of the crack front. In Figure 13, such a crack front is shown during the DCB test by displaying SDEG (overall scalar stiffness degradation). When SDEG in an element reaches critical value, it can no longer accumulate any damage or transfer stresses and it is removed from the simulation. It is visible that the crack propagates quicker in the central part of the specimen, which is another argument that methods that are based only on the visual observations of the crack growth in the side of the specimen may not be accurate for such types of materials. Also, one can observe gradual stiffness degradation behind the crack front, which is one of the assumptions that CZM utilizes.

**Figure 14:**
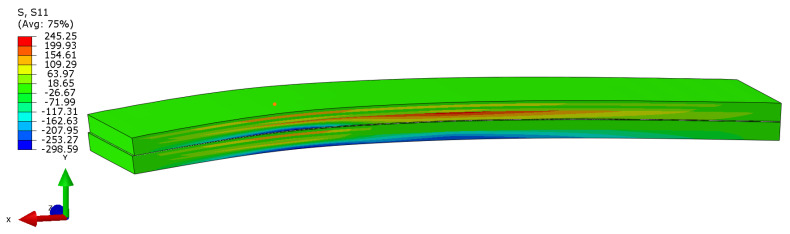
Numerical model of ENF test. The sliding of the upper and lower beams is visible in the left part of the image.

The numerical model for the ENF test was built in an analogical way to the DCB one. An important aspect characteristic of this one is introducing contact between layers that initially are not bonded due to PTFE film. This is visible in Figure 14 after the initially loaded upper and lower beams start to rub against each other. The contact defined included friction (friction coefficient was defined as 0.2 based on the literature) and allows separation after contact if needed. Due to the complexity of the model itself (cohesive elements layer and their degradation), the boundary conditions were defined directly on the model and not through the contact and modeling supports and loading nose as analytical rigid. This is a simplification (due to ignoring the fact that during the test the contact area is changing), but it influences the results only slightly while reducing the computational time drastically.

**Figure 15:**
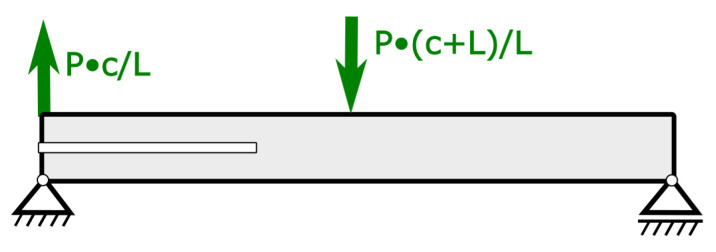
Forces acting on MMB specimen during the test with support points denoted. In the simulation displacement control of simulation was used and loading conditions were transposed to displacement ratio using auxiliary simulations.

Similarly, as in the case of the ENF test, a numerical model of the MMB test was limited to the specimen itself. Modeling the whole experimental setup through rigid bodies is possible but not efficient. The presented approach is usually used by other researchers as well (e.g., [40,41]). The MMB test is basically a three-point bending test with additional opening force on the end of the specimen transferred through the lever. Assuming L as the support span, c as a lever length and P being force generated by testing machine boundary conditions in terms of force and fixation are described in Figure 15. A more detailed description of the MMB test can be found in the introduction. By manipulating the length of the lever c, it is possible to obtain different mixicity ratios.

**Figure 16:**
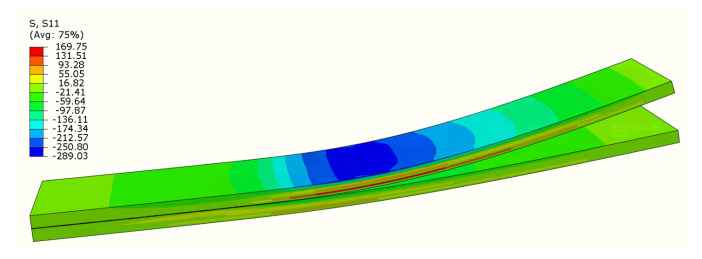
MMB specimen during the test (stress along specimen length).

Figure 16 shows the boundary condition that could be applied to the load-controlled simulation. However, for tested materials, this is not a sufficient approach, as a decrease and re-increase in the forces is expected. Thus, similarly to the previous models, displacement-controlled simulation is preferred. This approach, however, demands describing displacement between the end of the specimen ($\delta_{DCB})$ and placement of loading nose ($\delta_{ENF})$. To analyze this, auxiliary load-controlled simulations were carried out and it was found that these displacements are proportional to each other. For two analyzed cases, ratios $\frac{\delta_{ENF}}{\delta_{DCB}}$ were determined as 2.8 for mixicity ratio ϕ=33% and 1.82 for ϕ=45.5%. In Figure 16, MMB specimen is presented along with the stress distribution obtained from the model. The cohesive layer consists of elements with a size under 0.1 mm. This size was determined by comparing different sizes of mesh. However, the rest of the specimen mesh could be coarser. Thus, a cohesive layer was modeled as a separate part and connected to the rest of the specimen by ties. Due to this, it is possible to have different mesh sizes on the cohesive and metal/composite layers. This approach significantly decreases computational time.

**Figure 17:**
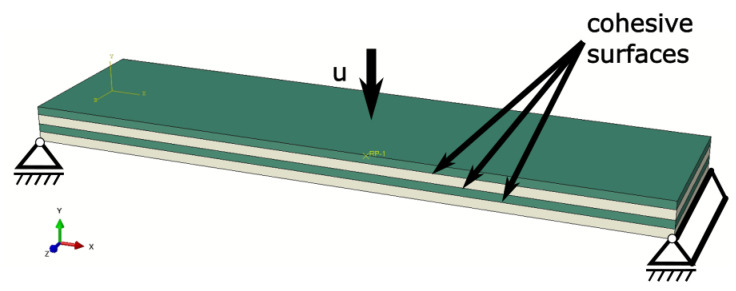
Assembly of model for three-point bending test with used boundary conditions and kind of implementation for the cohesive interface between metal (beige) and composite layers (green).

There is a case where the three-point bending specimen used in the experiment did not contain any initial delamination. Because of that, it is not certain where damage in the interface will show, as there are multiple metal–composite interfaces in the specimen. Due to this fact, another approach to the model interface was used—instead of cohesive elements, cohesive surfaces were introduced to stabilize the model, as with other approaches, the time of the simulation would be significantly longer. The idea of the model along with boundary conditions is presented in Figure 17. The material model that was obtained from other numerical models and experiments was used.

The main output of this model was the force–displacement characteristic and displacement map, as it can be easily compared with experimental results (see Section 3) from the testing machine and DIC system. Besides that, other outputs were also analyzed. Force–Displacement curves obtained from all models are presented in the next section along with experimental test curves.

## 3. Results

### 3.1. Building Interface Material Model Based on TESTS and FEA

**Figure 18:**
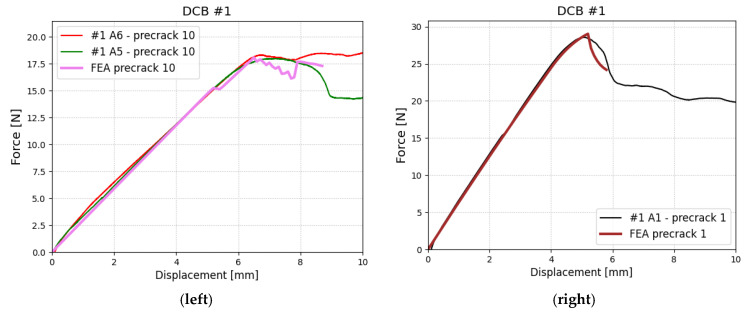
Results of DCB test for the exemplary length of precrack. (**left**) figure are experimental and numerical results for specimens with additional precrack 10; (**right**) with precrack 1.

In the case of DCB, the experimental test procedure of creating a precrack was not repeatable and resulted in an initial crack length between 61 and 70 mm. In order to use all obtained data, a numerical model was adapted for every precrack length. Then, the material parameters were chosen in such a way that the numerical model reflects the experimental results. In Figure 18, force–displacement relationships are shown for two edge cases—additional (to 60 mm) precrack length of 1 mm (the smallest observed) and 10 mm (the largest observed). All presented results in this section refer to the configuration denoted with #1 which is a subject of this paper.

**Figure 19:**
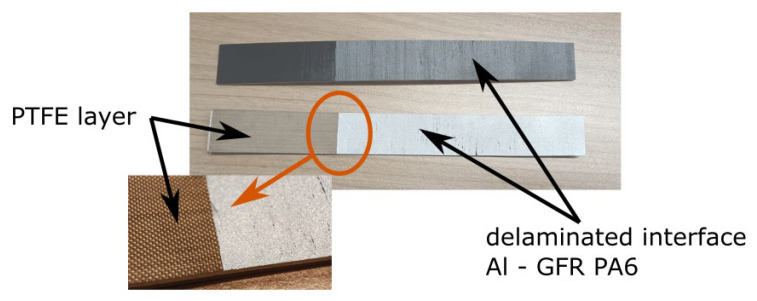
Delaminated specimen. On the left is the initial not bonded part with the PTFE layer.

Each specimen was analyzed postmortem, to check if the original bonding was of a good quality as an addition to previously run SEM analysis (as shown in Figure 5). In Figure 19, a fully delaminated specimen is shown (it was fully delaminated manually after the test was stopped). There is a distinctive difference between the area that was initially bonded and one that contains PTFE film. The determined values for mode I testing are $\sigma_{Ic}=10\;\mathrm{MPa}$ and $G_{IC}=0.5\frac{N}{\mathrm{mm}}$. The values were checked using 1 MPa resolution for $\sigma_{Ic}$ and 0.1 $\frac{N}{\mathrm{mm}}$ for $G_{IC}$.

**Figure 20:**
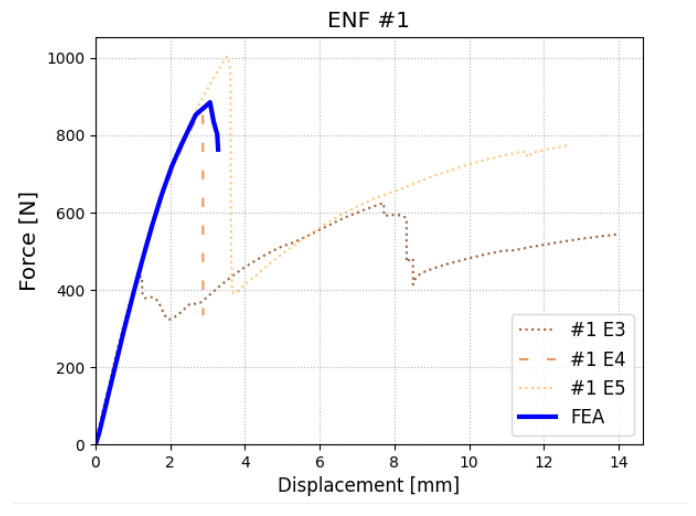
Results of ENF test—experimental and numerical results.

Experimental results and numerical models reflecting an ENF test are shown in Figure 20. In the case of this test, there were experimental problems with shearing in non-initially delaminated interfaces (as there are in total three interfaces of the metal–composite designed for research campaign specimens). All specimens where the problem occurs were discarded (an example in Figure 20 is shown, with the code E3). The behavior could be expected, as the numerical analysis shows high stresses in all three interfaces. Nevertheless, a numerical model was adjusted to successful specimens (E4, E5), and values for mode II were determined as $\sigma_{IIc}=30\;\mathrm{MPa}$ and $G_{IIC}=3.5\frac{N}{\mathrm{mm}}$. The values were checked using 1 MPa resolution for $\sigma_{IIc}$ and 0.1 $\frac{N}{\mathrm{mm}}$ for $G_{IIC}$.

**Figure 21:**
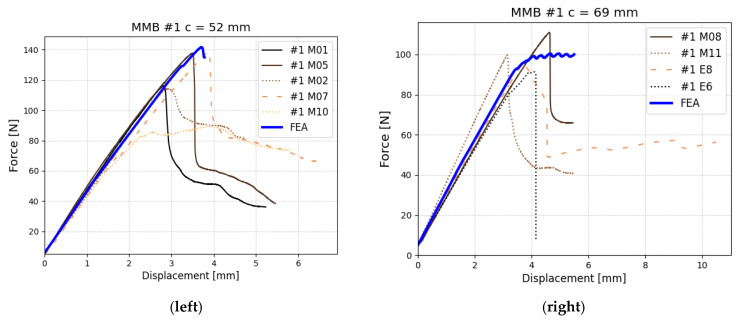
Experimental and numerical results for MMB tests with different mixicity ratios (**left**) for lever distance c = 52 mm, which is mixicity ratio ϕ=44.5%; (**right**) for lever distance c = 69.5 mm, which is mixicty ratio ϕ=33%.

Finally, we determined the B-K coefficient based on the MMB test, which was realized for two different mixicity ratios: ϕ=44.5% and ϕ=33%. Based on the specimens tested for these two ratios, the value of exponent coefficient m (also denoted as η) was set as 0.5. Unfortunately, mixicity ratios were not very distinct from each other, which causes very minor differences between a wide range of m values, making m determination less accurate. This should be enhanced in future usage of the proposed method. Nevertheless, for the assumed m value of 0.5, we obtain the best compatibility between the experiment and numerical results as presented in Figure 21 (left) (ϕ=44.5%) and Figure 21 (right) (ϕ=33%). The m value was checked with 0.05 resolution. In each case, six specimens were considered, but we did not include ones that had damaged piano hinges instead of the specimen.

### 3.2. Validation of Material Model—Three-Point Bending

**Figure 22:**
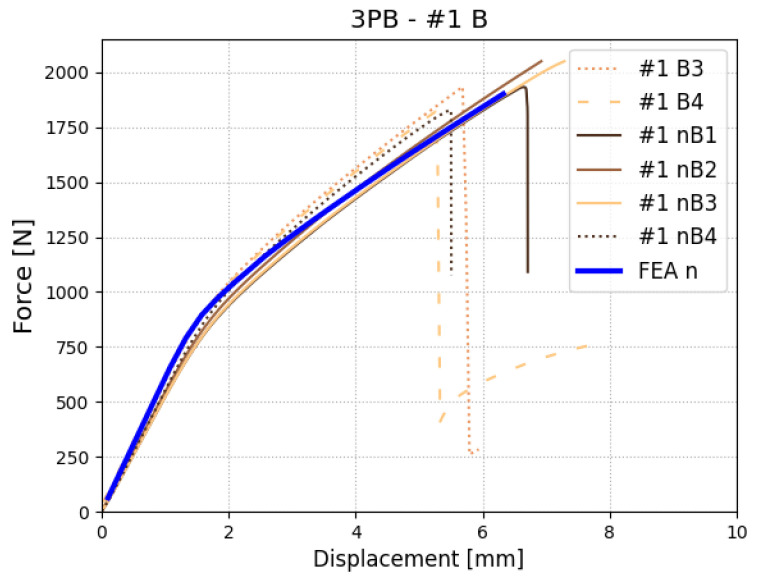
Force–displacement results for the three-point bending test—numerical (blue solid line) and experimental (specimens denoted with #1 and symbolic name).

**Figure 23:**
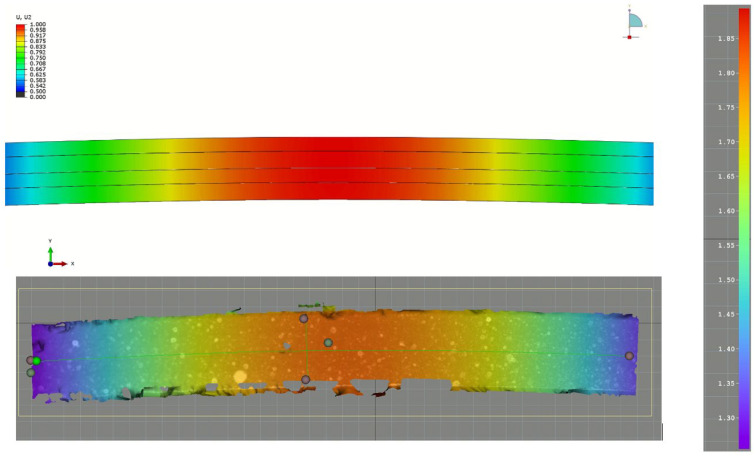
Comparison between displacement in finite element method and digital image correlation. The values differ due to other 0 definitions. However, relative displacements are comparable for both methods.

In this section, a comparison between the final numerical model and the experimental three-point bending test is shown. The numerical model utilized a material model developed from all previous tests. To validate its correctness, we compared force–displacement curves from FEA and experimental tests (see Figure 22). The developed model shows a good correlation both in the elastic part and also after damage is initiated. Maximum observed force/displacement lies between values obtained from the experiment. In Figure 23 comparison between displacement in finite element method and digital image correlation is shown.

**Figure 24:**
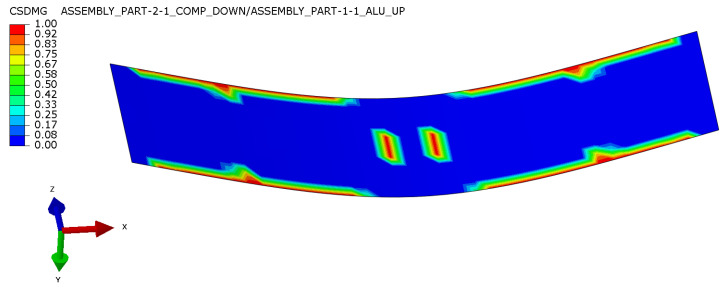
Damage parameter for a cohesive interface between metal and composite layer (one of) for specimen in three-point bending test.

Finally, output CSDMG (Abaqus damage variable for cohesive surfaces in general contact) showing damage in the interface layer was compared with the visual state of post-mortem specimens. In Figure 24, one can see damage localized in the edges around supports and in the middle of the width in the region where the load was applied—these areas were confirmed by post-mortem analysis. Due to the high interlaminar stresses at the edges, it is a source of the initiation of delamination and its importance should be considered in designing.

## 4. Recommendations and Conclusions

The method presented can be applied to other configurations or types of thermoplastic FMLs. We formulated some recommendations for future reuse of the method:

- As the method relies on adjusting material models in numerical simulations, basic material data should be, if possible, obtained from self-conducted tests (to provide more realistic data than one taken from data sheets), especially the Young modulus in both directions, as it has significant influence over final results.
- For similar reasons, the fiber content and direction should be controlled for all specimens. If any discrepancy is observed, it has to be compensated for during the numerical modeling process (fiber misalignment)
- During the experiment, the sides of the specimens should be covered with white paint, and the surface should be examined during the test to exclude specimens where multiple interface delamination occurs. The delamination progress can also be compared with the numerical simulations as another validation method.
- Because the DCB and MMB test-designed configuration should have similar stiffness in the upper and bottom beam, we note that it is crucial that the method is the type of the interface that is tested and not an exact configuration of the FML, as we determine material model for the interface. Because of that, our proposed configuration does not contain layers of metal on the top and bottom. This should also be taken into account when planning the technology for the adhesion of blocks or piano hinges.
- In the case of DCB, ENF and MMB specimens, an initial precrack should be created using preliminary loading. The length of the precrack should be ideally the same for every specimen, but if not, it should be measured and numerical models should be adjusted for every test with a different precrack.
- In the case of ENF and MMB tests, experiments can be stopped soon after the maximum value (e.g., after the first re-rise of the force after the maximum). In the case of the DCB test, the character of the force curve is different and the experiment should be stopped later (e.g., when the character of the post-peak curve is clear for the experimenter)
- The MMB test should be taken for at least two different values of mixicity rate—suggested values are 33% and 66%, but if they are experimentally problematic, as in our case, they may be adjusted. More mixcity ratios may be checked if the values for mode I and mode II are close to each other.
- The method was investigated in this paper with the assumption of bilinear CZM and B-K approach for mixed-mode, but it can be also applied to other variations of CZM or mixed mode. For more complex variations, more tests should be conducted, as more parameters have to be determined.
- We propose validation of the determined model by running of 3PB test because it is a simple, standard test. It is possible to use also other tests; however, they must include some sort of mixed-mode loading conditions.
- Numerical models can utilize both the cohesive elements or the cohesive surface approach, but the size of the elements may be crucial and should be investigated. Because simulations may be not very stable, we advise utilizing symmetry and using simplified boundary conditions defined on the specimens and not including supports and indenter with contact definition. Numerical simulations should be controlled by displacements.

By following the defined recommendations, it is possible to apply the proposed method to others similar to the tested materials. Further development of the method could lead to the development of an automatized way of conducting the process.

In the paper, the method for the determination of fracture parameters for thermoplastic FMLs was proposed. The method is based on performing five different tests:

- Tensile tests of constituent material and FML itself to determine elastic properties,
- DCB test and reflecting the numerical model for determination of fracture energy and initiation stress in mode I (opening of the interface)—elastic data based on the tensile test,
- ENF test and reflecting the numerical model for determination of fracture energy and initiation stress in mode II (shearing of the interface)—elastic data based on tensile test,
- MMB test and reflecting the numerical model for determination of fracture energy and initiation stress in mixed-mode I+II (opening and shearing of the interface)—elastic data based on tensile test, data for mode I and mode II fracture based on DCB and ENF tests, respectively,
- 3PB test and reflecting the numerical model for validation of the material model derived from previous tests and models.

**Figure 25:**
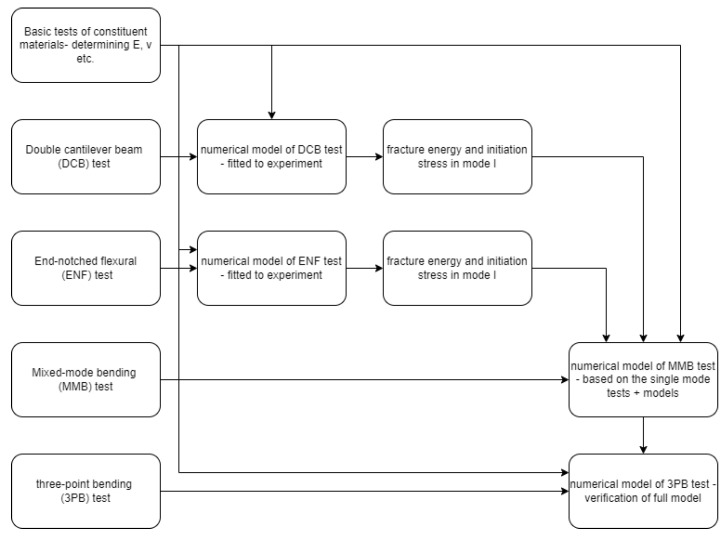
Flowchart of the proposed approach to determine the full material model for fracture of thermoplastic FML and its verification.

A summary of the method presented in the paper in the form of a flowchart is shown below in Figure 25.

A summary of the experimental results along with numerical values is shown below in Table 5. The results show good correspondence between results obtained from the experimental tests and numerical models. However, the future method should utilize more specimens per test to increase the certainty of the concluded values.

For the verification of the method, thermoplastic FML material was prepared and manufactured in the hot-press process. The material analyzed is based on aluminum (metal layers) and polyamide 6 reinforced by glass fibers (composite layer). Fracture parameters of the metal–composite were determined by applying the described method and are presented in Table 6.

**Figure 26:**
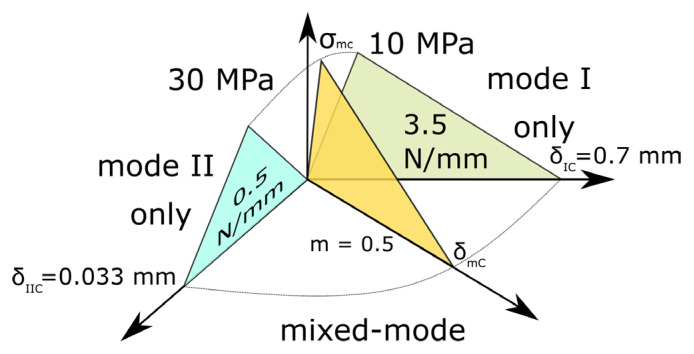
Graphical summary of the obtained fracture parameters of the interface in thermoplastic (polyamide 6) FML.

The obtained values (presented in Figure 26) can be now used to model mixed-mode fracture of such composite for arbitrary geometry following the cohesive zone model and B-K approach. The presented method can be applied also to other similar materials.

## Figures and Tables

**Table 1:** ASTM standards devoted to the mechanical investigation of interface fracture properties.

Fracture Mode	Standard Designation
Mode I	ASTM D5528 (static) [2]/D6115 (fatigue) [3]
Mode II	ASTM D7905 [4]
Mode I+II	ASTM D6671 [5]

**Table 2:** Summary of designed specimens for different kinds of tests.

Test	Width [mm]	Length [mm]	Initial Delamination [mm]
Tensile	20	200	0
3PB	20	180	0
DCB	20	180	60
ENF	20	160	60
MMB	20	180	60

**Table 3:** An important parameter of the ENF test.

Parameter	Value
Support span [2L] [mm]	80
Initial delamination length [mm]	60
Indenter speed [mm/min]	1
Indenter displacement [mm]	10 or 15
Support radius [mm]	5
Indenter radius [mm]	5

**Table 4:** Summary of parameters in three-point bending tests.

Parameter	Value
Support span [mm]	80
AE sensors span [mm]	120
Loading speed [mm/min]	2
Support radius [mm]	5
Stamp radius [mm]	5

**Table 5:** Summary of experimental peak forces and corresponding critical displacements for various tests conducted in this study.

		3PB	DCB Precrack 1	DCB Precrack 10	ENF	MMB ϕ = 44.5%	MMB ϕ =33 %
**Peak Force [N]**	Experiment	1935.1± 104	28.6 ± 0	18.1 ± 0.2	933.8 ± 98.5	138.2 ± 0.8	99.5 ± 8.3
	FEA	1901.2	29.0	18.1	885.4	141.8	98.6
**Peak Force Displacement [mm]**	Experiment	6.21 ± 0.84	5.06 ± 0	6.95 ± 0.37	3.19 ± 0.49	3.69 ± 0.27	3.90 ± 0.61
	FEA	6.33	5.20	6.50	3.07	3.70	3.99

**Table 6:** Fracture parameters obtained for tested configuration of thermoplastic FML. Below the values, the source for the value is shown. All values were obtained from adjustment to experimental values in a particular numerical model and then verified in another test.

$\sigma_{Ic}\;\left[MPa\right]$	$\sigma_{IIc}\;\left[MPa\right]$	$G_{I}\;\left[N/mm\right]$	$G_{II\;}\left[N/mm\right]$	$m\;\left[-\right]$
10	30	0.5	3.5	0.5
**DCB (FEA)**	ENF (FEA)	DCB (FEA)	ENF (FEA)	MMB (FEA)

## Data Availability

The datasets presented in this article are not readily available because the data are part of an ongoing study. Requests to access the datasets should be directed to corresponding author.

## Nomenclature

MVF	Metal volume fraction
B-K	Benzeggah–Kenane equation for mixed mode
DCB	Double cantilever beam
ENF	End-notch flexural
FML	Fiber–metal laminate
MMB	Mixed-mode bending
PA6	Polyamide 6 thermoplastic material
PTFE	Polytetrafluoroethylene
3PB	Three-point bending
CZM	Cohesive zone model
δ	Displacement
ϕ	Mixicity ratio
σ	stress, traction
L	Length of the MMB specimen between supports
c	Length of the lever in the MMB test
$\sigma_{Ic}$	Maximum (critical) traction in mode I (opening)
$\sigma_{IIc}$	Maximum (critical) traction in mode II (shearing)
$G_{IC}$	Fracture energy in mode I (opening)—area under traction–separation curve
$G_{IIC}$	Fracture energy in mode II (shearing)—area under traction–separation curve
$G_{TC}$	Fracture energy in mixed-mode I+II
m	The exponent of the Benzeggah–Kenane formula for mixed-mode loading
